# TOBACCO USE AND COGNITIVE FUNCTION AMONG OLDER ADULTS IN MEXICO AND BRAZIL

**DOI:** 10.1093/geroni/igad104.2431

**Published:** 2023-12-21

**Authors:** Jaqueline Avila, Natalia Gomes Goncalves, Joseph Saenz, Claudia Suemoto, Rebeca Wong

**Affiliations:** University of Massachusetts Boston, Boston, Massachusetts, United States; University of Sao Paulo Medical School, Sao Paulo, Sao Paulo, Brazil; Arizona State University, Phoenix, Arizona, United States; Universidade de São Paulo, São Paulo, Sao Paulo, Brazil; UTMB, Galveston, Texas, United States

## Abstract

Older adults in Brazil and Mexico have high lifetime tobacco exposure, which is an important modifiable dementia risk factor. They also have lower levels of education, which is associated with lower cognitive reserve. This study examines how tobacco use is associated with cognition and how this association differs by education among older adults in Brazil and Mexico. Data included adults 50 and older in the 2015 waves of the Mexican Health and Aging Study (MHAS, n=14,018) and the Brazilian Longitudinal Study of Aging (ELSI, n=9,409). The outcome was a global cognitive score, calculated with standardized cognitive tasks. Tobacco use was defined as current, former, and never smoker. Linear regression models were used to test differences in cognitive function by tobacco use, adjusted by sex, educational level, rural/urban area, chronic disease and included the interaction between smoking and education. Current and former smokers represented 17% and 37% of the sample in Brazil and 13.4% and 27.4% in Mexico, respectively. Current smokers in both countries were more likely to be men and be younger. However, current smokers in Brazil were more likely to have lower educational levels whereas those in Mexico were more likely to have 8+ years of education. In Brazil, current smokers had lower cognitive scores than never smokers (B= -0.06; 95%CI: -0.11; -0.01). In Mexico, current and former smokers had higher cognitive scores than never smokers (B=0.09 ; 95%CI: [0.05; 0.13] and B=0.10 [0.07;0.13], respectively). Cognitive scores for current smokers were significantly lower as education increased in Mexico.

